# Can the COVID-19 pandemic boost the global adoption and usage of eHealth solutions?

**DOI:** 10.7189/jogh.10.0203101

**Published:** 2020-12

**Authors:** Dalibor Stanimirović, Vedrana Matetić

**Affiliations:** 1 National Institute of Public Health, Trubarjeva, Slovenia; 2 National Institute of Public Health, Ljubljana, Slovenia

**Figure:**
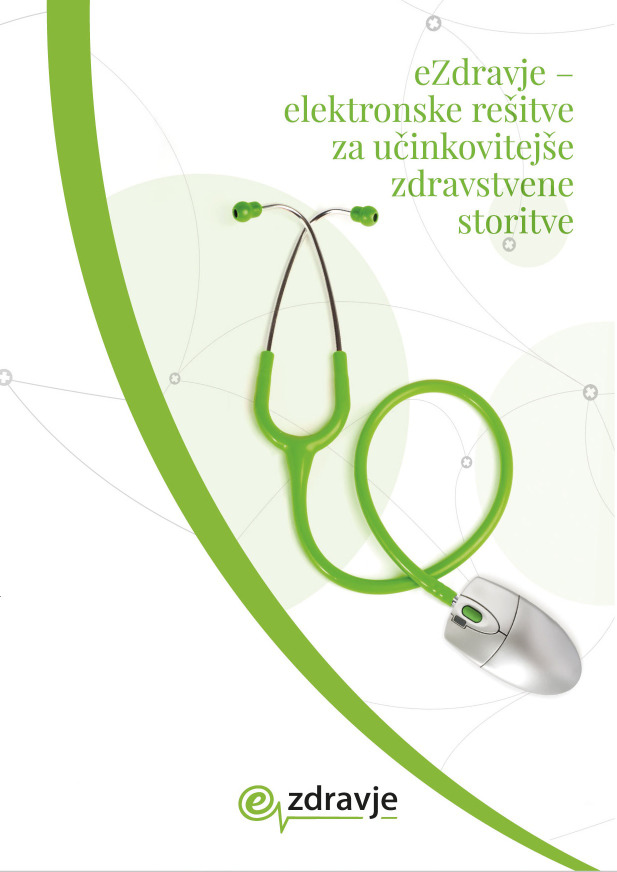
Photo: Poster from the National Institute of Public Health, Slovenia.

The public health care system in Slovenia has been struggling with numerous challenges in recent years, due to various systemic and socio-economic circumstances and unfavourable public health trends [1]. The system has been facing management issues and a shortage of resources on one hand, and often outdated and inappropriate legislation on the other. To confront these challenges and ensure the sustainability of the public health care system, profound and wide-ranging changes to the current health care arrangements are needed.

In view of that, the digitalisation has been deemed essential for innovation and setting up a more efficient and successful health care system. The term “digitalisation” is in this text defined as a comprehensive introduction of information and communications technology (ICT) solutions into the health care system operational and business processes. In international strategic documents, ICT represents one of the essential instruments for achieving the improved medical treatment of patients and ensuring timely monitoring of all operating parameters in the health care system [2]. The most recent Slovenian strategy document, ie, “Resolution of the national healthcare plan 2016-2025”, lists several specific goals in the area of ICT in health care. In compliance with the EU documents emphasising the efficiency, accessibility, and flexibility of health care systems, it primarily highlights the implementation of unified and efficient ICT solutions as an overarching strategic objective. Such ICT solutions would provide relevant data for the medical treatment of patients and support evidence-based decision-making [3]. Dependable medical, financial, and administrative data would enhance the planning and management of both individual health care providers and the health care system in general [4]. The research reveals that successful health care digitalisation projects have enormous strategic importance for further advancing the health care system and the far-reaching impact on economic growth and social development [5].

## DIGITALISATION IN PROCESS AND THE COVID-19 OUTBREAK

The Slovenian health care digitalisation project (eHealth), following the national, European, and World Health Organization guidelines on ICT in health care, is one of the key long-term goals of the public sector in Slovenia [6]. The Slovenian eHealth in its current form covers digital solutions such as electronic prescription (ePrescription), electronic appointment (eAppointment), Central Registry of Patient Data (CRPD, containing specialist reports, microbiology reports, discharge letters, ambulatory exam reports, vaccinations, and other patient records), and Patient Portal, to name just a few of the most important. Then, of course, there is a whole range of back-end infrastructure and network platforms that actually enable the use of eHealth solutions, but they are not generally user-oriented and therefore they are not specifically outlined in this text. Considering the events since the publication of the first strategic document involving health care digitalisation in 2005, the implementation of eHealth solutions from 2016 onwards represents an important milestone in the history of the Slovenian health care system. This is supported by statistical data and different evaluations carried out by national and international institutions. The percentage of ePrescriptions among all drug prescriptions in the year 2019 was above 92% (calculated as a monthly average). In absolute figures, this represents more than 1 150 000 ePrescriptions per month. Similarly, the share of eAppointment referrals averaged more than 95% per month in the last year (more than 300 000 referrals per month). The number of health-related documents sent by health care providers into the CRPD is rising steadily. The Patient Portal had more than 768 000 visits in 2019, compared to 548 000 visits in 2018. **Table 1** shows the cumulative growth in the use of eHealth solutions in Slovenia on an annual basis since their introduction into the health care system in 2016 until the end of 2019. The cumulative figures for this year are understandably not yet available. Over the years, it is possible to see a constant growth in the use of eHealth solutions, and according to interim data in 2020, these figures are likely to increase in absolute or relative terms this year, despite the distressing and unpredictable public health situation due to the COVID-19 epidemic.

**Table 1:** Annual growth in the use of eHealth solutions in Slovenia, 2016-2019.

		2016	2017	2018	2019
eAppointment	Number of eReferrals	241 379	2 509 518	3 564 993	3 946 878
	% of all Referrals	42.96	84.71	95.11	93.92
ePrescription	Number of ePrescriptions	12 326 845	13 095 808	13 867 192	13 895 517
	% of all Prescriptions	87.23	88.73	92.33	93.47
CRPD	Number of documents	3 180 704	6 436 900	9 411 132	15 201 309
Patient Portal	Number of visits	669	262 012	548 512	768 255

CRPD – Central Registry of Patient Data

**Figure 1:**
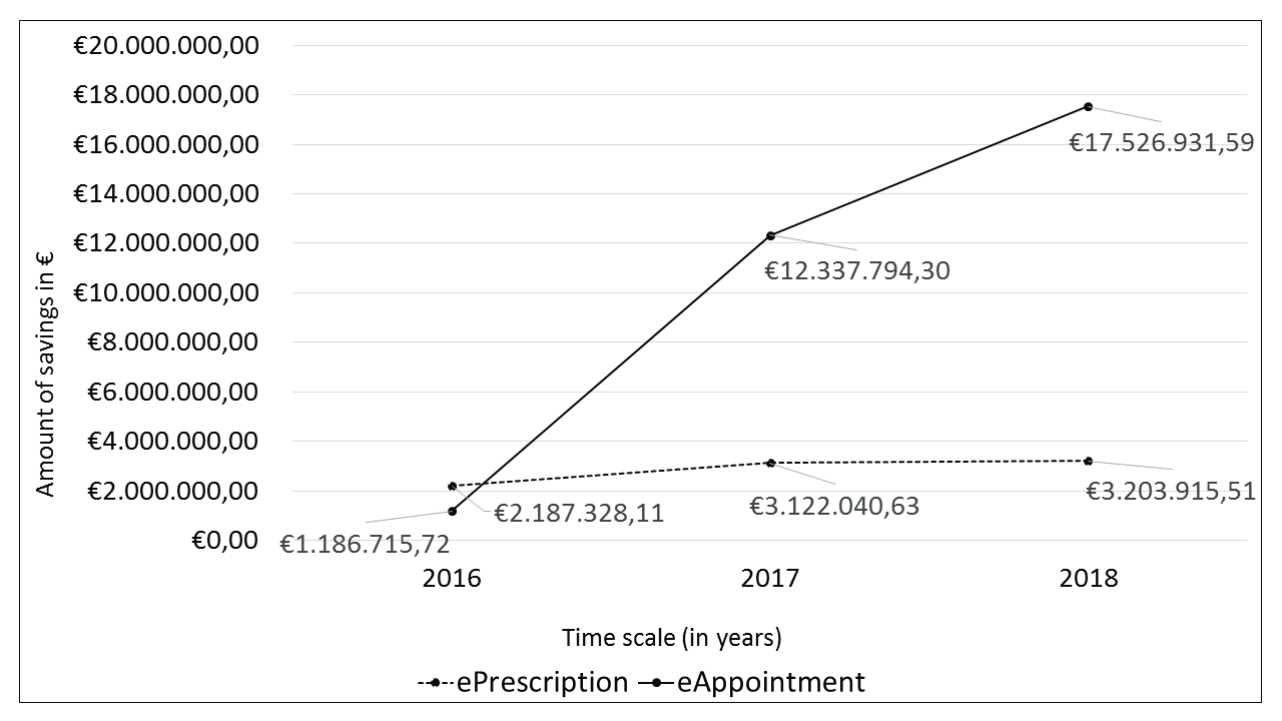
Estimated savings from ePrescription and eAppointment, 2016-2018.

An evaluation by the Ministry of Public Administration for the 2016–2018 period shows that the usage of eHealth solutions (ePrescription and eAppointment) resulted in significant savings in the health care system; the Ministry of Public Administration’s estimate of the accumulated savings is approximately €40 million [7]. In addition to financial savings, the evaluation highlights other systemic benefits of ePrescription and eAppointment, such as streamlined and more effective treatment processes, simplification of procedures for patients, greater standardization, quality and safety of collected health data, consultations between general practitioners and specialists, lower administrative costs, availability of data for analysis and research, etc. The annual dynamics of savings from the evaluation of the Ministry of Public Administration is illustrated in **Figure 1**.

The Digital Economy and Society Index (DESI) of the European Commission is a compound index, which encapsulates pertinent indicators on digital performance and monitors the developments in digital competitiveness of EU Member States. DESI Report 2019 indicates a big breakthrough in the development and usage of eHealth services in Slovenia, ranking Slovenia in 6th place in the EU [8]. Slovenia's position is well above the EU28 average, and it also ranks better than many countries with comparable GDP (or even higher) and a comparable population.

Digital solutions for monitoring quality and safety in hospitals are also relatively well developed. In 2002, the Ministry of Health set up a system for monitoring warning events and reporting by health care providers. In accordance with the requirements, hospitals have introduced internal digital solutions in the form of online self-assessment questionnaires, which enable monitoring of quality and safety indicators, as well as appropriate actions in the event of identified deviations. The development of a national web portal is also under way, which will enable patients to report perceived deviations in the quality or safety of their own medical treatment. Due to the COVID-19 epidemic, the project was unfortunately suspended in April 2020.

Specialized online expert systems, which enable advanced decision-making support and predictive analytics with the help of artificial intelligence, are used in individual areas. However, this approach is not widespread in Slovenia and is not routinely used by health care providers. Expert systems, which are usually used in combination with decision-support algorithms and smart devices, are mostly used in monitoring and interpretation of simultaneous analyses of a large number of data in specific areas, where this is substantively and technologically feasible (COVID-19 patients, chronic patients, monitoring of cancer patients, clinical chemistry, etc.). Nevertheless, it should be emphasized that this branch of digital solutions is only in its infancy in Slovenia and that the main initiatives for the development of such approaches have come from national and international research projects in recent years.

As we can see, a multilateral analysis of the maturity, usage, and efficiency of eHealth puts Slovenia high on the list of the most successful countries in the area of eHealth. But after the first COVID-19 case was discovered in Slovenia at the beginning of March 2020 and the epidemic was declared several days later, the questions of the benefits of eHealth during this situation began to appear. Although several analyses and assessments of eHealth were performed in the past, there has been no observation or evaluation of the potential benefits of eHealth solutions in the context of the epidemic. Remarkably, many aspects of the development and use of digital solutions in health care have been studied so far, including those of a peripheral nature, which often manifest barely sufficient importance for a legitimate research interest. Nevertheless, it is possible to detect the lack of research interest in such a vital area as the potential benefits of digital solutions in situations like the COVID-19 epidemic. In accordance with these starting points, we provide below an outline of the course of the COVID-19 epidemic in Slovenia and an overview of socio-economic consequences and response measures at the national and EU level. And, most importantly from the point of view of our manuscript, we present an analysis regarding the role and value of eHealth solutions for health care professionals and patients during the COVID-19 epidemic in Slovenia.

The first COVID-19 infection case in Slovenia was discovered on 4 March 2020. The epidemic was officially declared on 12 March. The government quickly introduced a series of restrictive measures to fight the COVID-19 outbreak. Due to the relatively positive developments, the measures were gradually phased out at the beginning of April. The government declared the COVID-19 epidemic officially over on 31 May. The total number of cases was 3312 on 9 September. The number of deaths due to COVID-19 was estimated at 135. Although the epidemic was declared over, the virus is still present and the epidemiologists are discussing the possibility of a second (or third) wave of COVID-19 by the end of the year. Protective measures such as social distancing and mask wearing are still required. The epidemic and the accompanying measures had severe consequences on the Slovenian economy and health care and other social subsystems. To lessen the impact, the government adopted several regulatory measures, the total value of which exceeds EUR 6 billion [9]. Still, it is already obvious that the epidemic will have profound and lasting consequences.

Generally speaking, we could say that the socioeconomic situation in the EU is even worse. The European Commission is proposing to harness the full potential of the EU budget for the mobilisation of investments and financing of key areas for the recovery of the EU Member States. The Commission is planning on activating an emergency European Recovery Instrument amounting to €750 billion, together with the three important safety nets for workers, businesses, and sovereigns amounting to a package worth €540 billion, thus reaching €1290 billion of targeted support measures for the recovery of EU Member States [10]. These funds will be channelled through the EU budget to Member States for key areas such as reinforcing health care and social systems, and supporting the green and digital economy, with the final goal of ensuring the sustainable development and more stable socioeconomic foundations of EU Member States. If the crisis continues for longer or if there is a second wave of the pandemic, there will be additional funds for appropriate measures, as emphasised by the representatives of the European Commission.

Considering the digitalisation aspect, one should ask what the role and value of eHealth solutions for health care professionals and patients were during the COVID-19 pandemic. The daily operation of the health care system came to a halt during the epidemic, the exception being emergency procedures and the treatment of oncological patients. The work of health care professionals was extremely difficult due to the new treatment protocols and the risk of infection. On the other hand, patients kept in-person visits to health care institutions to a minimum, due to a fear of infection, but also because of the changed treatment practice and various limitations imposed by health care institutions. eHealth and its various solutions (ePrescription, eAppointment, Patient Portal, CRPD) suddenly became the only way to provide quick, efficient, and safe health care services and to ensure appropriate communication, both internally between health care professionals and externally between health care professionals and patients. After the initial shock of the epidemic, the interest of health care professionals and patients in using eHealth solutions soared overnight and the reported learning curve flattened surprisingly. Healthcare professionals started using eHealth solutions more intensively, due to the extraordinary circumstances and other inherent factors, but also due to pressure from patients. On the other hand, patients have shown the greatest interest in monitoring their health and well-being through health-related documents accessible via the Patient Portal and CRPD.

**Figure 2:**
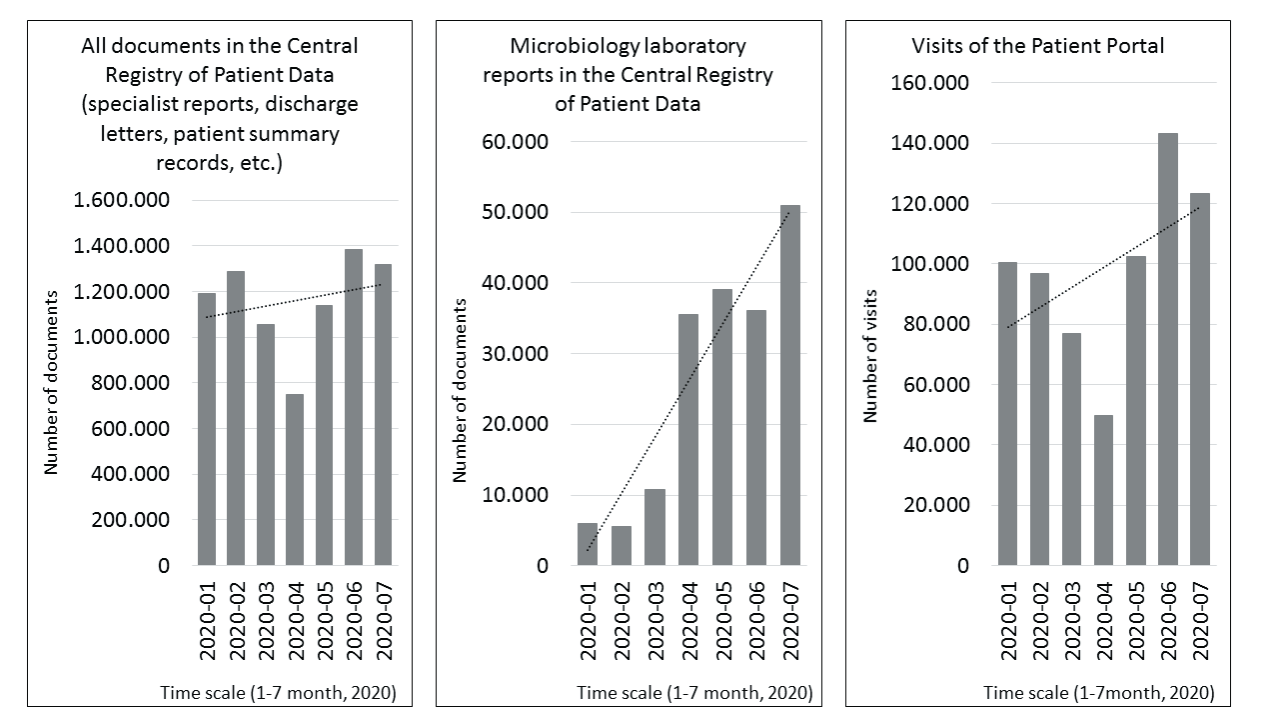
Growth in the use of individual eHealth solutions in Slovenia during the COVID-19 epidemic and immediately thereafter.

Statistical data supports the qualitative findings mentioned above. The CRPD and the Patient Portal in particular experienced a significant increase in usage during the lockdown period and immediately thereafter (**Figure 2**). However, other eHealth solutions, despite the very limited operation of the health care system in most areas and restricted patient admission, have maintained a relatively stable level of use, which also confirms their critical importance for the medical treatment of patients and the overall functioning of the entire health care system.

Experience from the first wave of the COVID-19 epidemic has shown that eHealth solutions have a very important role in such situations. Usage statistics have clearly shown that both health care professionals and patients recognise the many benefits of eHealth in unexpected and crisis circumstances that affect the health care system. Furthermore, the advantages of eHealth solutions for health care professionals and patients have proved to have an even greater impact in such conditions than in “normal” circumstances.

## CONCLUSIONS

It seems that the COVID-19 epidemic has done more to raise awareness and usage of eHealth solutions in a very short period of time than any other initiative before, be it of a political, legislative, administrative, or financial character. Given this alarming fact, it should be thoroughly examined and discussed what we did wrong, or what we did not do right, in having failed to intensify the use of eHealth solutions and convince users of the manifold benefits offered by digital solutions in the pre-pandemic era. The reasons for this undoubtedly go back to the lack of political will, insufficient stakeholder commitment, the absence of clearly defined sectoral policies and compelling goals for users with different motivating rationales, and a lack of training and education of users (health care professionals and patients). In addition to the outlined factors, the wide-ranging advocacy of eHealth and digitalisation, which is one of the fundamental principles in promoting national public health initiatives, has certainly failed.

If so, perhaps this epidemic (pandemic) may well mark a turning point in the perception of digitalisation as not only one of the crucial drivers for the development and promotion of public health, but also as an indispensable enabler in efforts to exploit existing health care system capacities and potentials, and empower patients in national and international public health crises, such as the present and probably all subsequent epidemics (pandemics).
